# Papular Umbilicated Granuloma Annulare in a Patient With Systemic Lupus Erythematosus

**DOI:** 10.7759/cureus.47600

**Published:** 2023-10-24

**Authors:** Maho Matsuo, Hirofumi Niwa, Hiroaki Iwata

**Affiliations:** 1 Dermatology, Gifu University, Gifu, JPN

**Keywords:** systemic lupus erythematosus, palisading granulomas, cutaneous vasculitis, histopathology (hp), granuloma annulare

## Abstract

Granuloma annulare (GA) is characterized by palisading granuloma, which is histopathologically distinguished by histiocytes arrayed in a palisade configuration encircling insoluble entities associated with degenerated collagen fibrils. The present case demonstrated multiple cutaneous papules showing palisading granuloma in a patient with SLE. A 39-year-old woman has been taking oral prednisolone daily, hydroxychloroquine sulfate, and belimumab for systemic lupus erythematosus (SLE). A few papules appeared on the lateral side of the left arm and gradually increased around both sides. Physical examination found multiple firm skin-colored papules ranging in diameter from 2 to 3 mm on both forearms. Some of the papules had umbilicated tops. Histopathological examination showed degenerated collagen fibers with mucin deposition surrounded by histiocyte infiltrates in the dermis. These findings are characteristic of palisading granuloma. There are several GA variants, such as generalized, subcutaneous, and perforating GA. We considered several possibilities of the mechanisms underlying characteristic histological changes; atypical generalized GA variants, dermatofibroma, and granuloma associated with cutaneous vasculitis. We made the final diagnosis of papular umbilicated GA in the context of SLE.

## Introduction

Palisading granulomas are typically observed in granuloma annulare (GA), necrobiosis lipoidica, and rheumatoid nodules [1]. Histologically, they are characterized by histiocytes arranged in a palisading pattern around insoluble substances related to degenerated collagen fibrils. GA is a cutaneous reaction of unknown cause, characterized by palisading granuloma [2]. We treated a patient with systemic lupus erythematosus (SLE) who demonstrated multiple cutaneous nodules showing histologically palisading granuloma. In this case, our clinical diagnosis of dermatofibroma did not correspond to the histological diagnosis of GA. We discuss the differential diagnoses for this case in the context of this discrepancy.

## Case presentation

**Figure 1:**
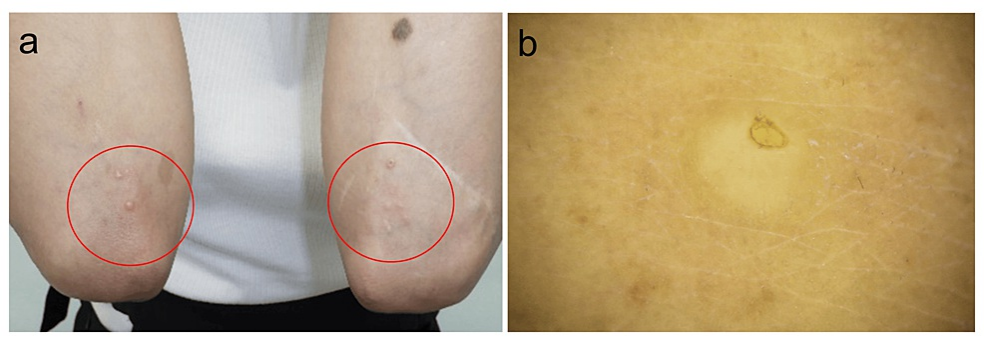
Clinical and dermoscopic features (a) Multiple papules are observed on both forearms. (b) Some of the papules are associated with umbilications (arrow). (c) A dermoscopic examination of the lesions shows a central white patch.

A 39-year-old woman had been diagnosed with SLE 18 years earlier. She has been taking 7 mg of prednisolone daily and 200 mg and 400 mg of hydroxychloroquine sulfate on alternating days. Additionally, she had been receiving weekly injections of belimumab. Hydroxychloroquine sulfate and belimumab had been introduced two years earlier to address the worsening of the SLE. After she started these medications, her clinical manifestations, including arthritis, oral ulcers, hair loss, vision problems, and psychiatric symptoms were well controlled. A few papules appeared on the lateral side of her left arm one month prior to dermatology referral and gradually increased around both sides. Physical examination found multiple firm skin-colored papules ranging in diameter from 2 to 3 mm on both forearms (Figure 1a).

**Figure 2:**
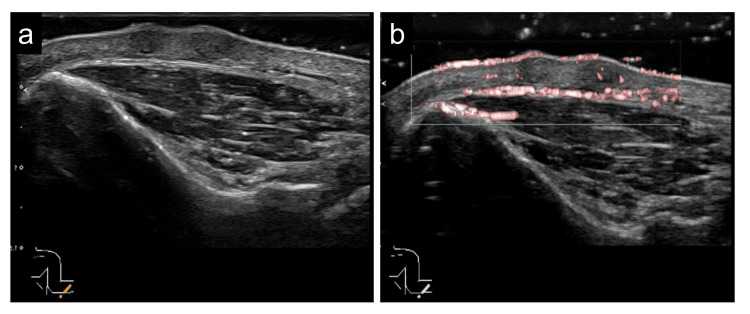
Echography findings (a) Echography shows a flattened, vaguely bordered, homogenous hypoechoic area. (b) Doppler imaging shows a hypoechoic area surrounded by an area of increased blood flow.

Some of the papules were associated with umbilications (Figures 1a, 1b, arrow). A dermoscopic examination showed central white structureless areas but no delicate pigment network (Figure 1c). Echography showed a flattened, vaguely bordered, homogenous hypoechoic area surrounded by increased blood flow (Figures 2a, 2b).

**Figure 3:**
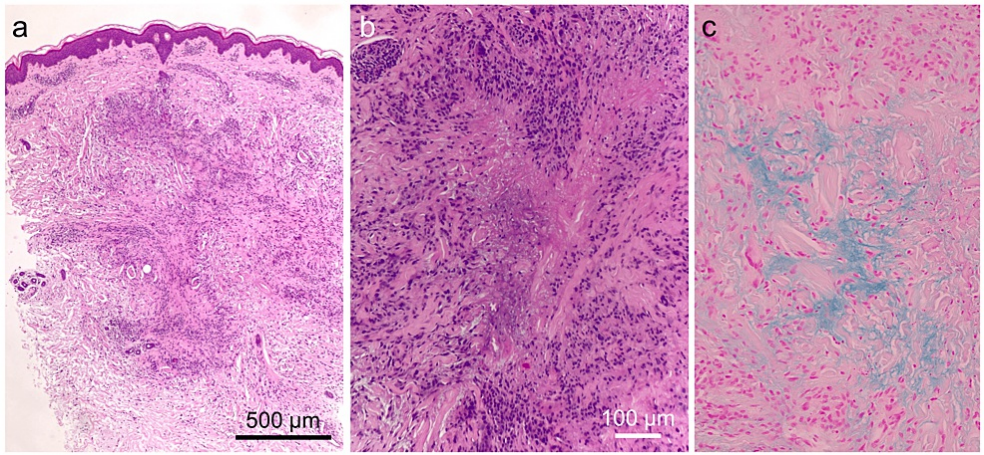
Histopathological findings (a) Histopathological examination shows central collagen degeneration with mucin deposition associated with inflammatory infiltrates. (b) A palisade of lymphocytic and histiocytic infiltrates surrounds degenerated collagen fibers. (c) Alcian blue stain demonstrates mucin deposits at the center.

There was no evidence of calcification. Blood tests showed leukocytes at 6.15x10³ cells/µL, platelets at 242x10³ cells/µL, CK at 41 IU/ml, IgG at 1174 mg/dL, CH50 at 43.6 IU/ml, C3 at 65 mg/dL, and C4 at 13 mg/dL. The antinuclear antibody titer was 1:160 (speckled type). Anti-ds-DNA antibodies had been controlled within the normal range before the onset of the cutaneous nodules, but our examination found them elevated to 67 IU/ml (normal: <12 IU/ml). Anti-ds-DNA antibody titer levels gradually rose from six months earlier, but the clinical manifestations were still well controlled. Myeloperoxidase anti-neutrophil cytoplasmic antibody (ANCA), PR3-ANCA, and rheumatoid factor were negative. Histopathological examination showed lymphocyte-dominated inflammatory infiltrates around vessels in the upper dermis. The epidermis showed slight acanthosis and basal melanosis. Additionally, radially degenerated collagen fibers with mucin deposition detected by Alcian blue staining were surrounded by histiocyte infiltrates in the dermis (Figures 3a, 3b, 3c).

There were no foam cells or Touton giant cells. Polarizing microscopy found no anisotropic compounds or structures. Additionally, tuberculosis was excluded by Ziehl-Neelsen staining and interferon-gamma release assays. These findings are characteristic of palisading granuloma. There was no obvious vasculitis in the dermis. The papules were diagnosed as papular umbilicated GA.

## Discussion

In the present case, our initial clinical diagnosis was multiple dermatofibromas, but the histology demonstrated a typical palisading granuloma. The histology showed a granuloma associated with mucin and degenerated collagen fibers, suggesting a so-called blue granuloma [3]. To address the discrepancy between the clinical and histological diagnoses, we considered several possible mechanisms that might underlie the characteristic histological changes. Classical GA is typified by erythematous plaques displaying a characteristic ring-like configuration along with granulomatous inflammation. Nevertheless, a spectrum of GA variants exists, encompassing generalized, subcutaneous, papular umbilicated, and perforating GA [4]. The generalized variant is characterized by diffuse papules on the extremities. Additionally, Barbieri et al. reported that GA is associated with several autoimmune disorders, including SLE, based on a population-based cohort study [5]. Particularly, there are several reports that interstitial GA is occasionally associated with SLE [6]. The histological findings differ from interstitial GA in this case. In the present case, umbilications were observed on the top of some papules. Given this evidence, papular umbilicated GA was the first possible diagnosis in this instance.

Second, multiple eruptive dermatofibromas are widely recognized in patients with connective tissue disorders such as SLE or in patients receiving immunosuppressive therapy [7]. Our initial suspicion revolved around the presence of multiple dermatofibromas or calcifications stemming from the SLE condition. Generally, the diagnosis of a prototypical dermatofibroma case poses no substantial challenge. The histopathological attributes deviated from the norm in the present case. In addition to common fibrous histiocytoma, numerous dermatofibroma variants, including the palisading type, have been described [8,9]. Although not a variant, a case of GA mimicking dermatofibroma was reported [10]. In this case, the clinical and dermoscopic findings were unable to be distinguished from dermatofibroma, but the histological features corresponded to typical GA. The histological palisading variant of dermatofibroma was a differential diagnosis in our case. According to previous reports, palisading dermatofibromas histologically show nuclear palisading areas in parallel rows that resemble Verocay bodies at the center and typical dermatofibroma at the periphery. These histological features were not observed in the present case; however, typical histological features of GA were observed.

Third, cutaneous nodules on the elbows showing palisading granulomas have been reported in Churg-Strauss syndrome and rheumatoid arthritis [11-13]. In such cases, small vessel vasculitis is associated with palisading granulomas. This condition is suspected of starting with leukocytoclastic vasculitis that results in palisading granulomas with collagen degeneration [11]. The histological features differ during the disease course. Many different disease names have been reported, such as “palisaded neutrophilic granulomatous dermatitis,” “necrobiotic granuloma,” and “interstitial granulomatous dermatitis with cutaneous cords and arthritis” [12,14]. These nodules commonly occur in patients with systemic vasculitis and connective tissue diseases including SLE. Additionally, the elbows, knees, and other extremities are commonly affected because of external pressure. The pathogenesis of palisading granuloma is assumed to be prolonged recurrent vasculitis, and external forces also contribute to the granulomatous changes.

Other than these three possibilities, we should consider drug-induced GA if the patient is taking certain medications. It is known that several drugs, such as allopurinol, amlodipine anti-TNF-α antibodies, and immune checkpoint inhibitors cause drug-induced GA [15,16]. However, the present patient had not taken additional drugs for at least the previous six months.

## Conclusions

In this case, our initial clinical diagnosis did not correspond to the histological diagnosis. Previous studies showed many variants of GA, so we made the final diagnosis of a rare variant of papular umbilicated GA associated with SLE. We will follow the patient carefully to observe whether the eruptions become widespread.
